# Illegal births and legal abortions – the case of China

**DOI:** 10.1186/1742-4755-2-5

**Published:** 2005-08-11

**Authors:** Elina Hemminki, Zhuochun Wu, Guiying Cao, Kirsi Viisainen

**Affiliations:** 1 Research professor, National Research and Development Centre for Welfare and Health STAKES, P.O. Box 220, 00531 Helsinki, Finland; 2 Associate professor, School of Public Health, Fudan University, P.O. Box 250, 200032 Shanghai, China; 3 Senior researcher, International Institute for Applied Systems Analysis, 2361 Laxenburg, Austria; 4 Senior researcher, National Research and Development Centre for Welfare and Health STAKES, P.O. Box 220, 00531 Helsinki, Finland

**Keywords:** Asia, China, population policy, illegal pregnancy, abortion, sex ratio

## Abstract

**Background:**

China has a national policy regulating the number of children that a woman is allowed to have. The central concept at the individual level application is "illegal pregnancy". The purpose of this article is to describe and problematicize the concept of illegal pregnancy and its use in practice.

**Methods:**

Original texts and previous published and unpublished reports and statistics were used.

**Results:**

By 1979 the Chinese population policy was clearly a policy of controlling population growth. For a pregnancy to be legal, it has to be defined as such according to the family-level eligibility rules, and in some places it has to be within the local quota. Enforcement of the policy has been pursued via the State Family Planning (FP) Commission and the Communist Party (CP), both of which have a functioning vertical structure down to the lowest administrative units. There are various incentives and disincentives for families to follow the policy. An extensive system has been created to keep the contraceptive use and pregnancy status of all married women at reproductive age under constant surveillance. In the early 1990s FP and CP officials were made personally responsible for meeting population targets. Since 1979, abortion has been available on request, and the ratio of legal abortions to birth increased in the 1980s and declined in the 1990s. Similar to what happens in other Asian countries with low fertility rates and higher esteem for boys, both national- and local-level data show that an unnaturally greater number of boys than girls are registered as having been born.

**Conclusion:**

Defining a pregnancy as "illegal" and carrying out the surveillance of individual women are phenomena unique in China, but this does not apply to other features of the policy. The moral judgment concerning the policy depends on the basic question of whether reproduction should be considered as an individual or social decision.

## Introduction

A current widely accepted principle is that it is the parents' – or the woman's – societal right to have children: as many as they want and are capable of having. Restrictions to this right include age limits in marriage, forbidding marriages involving close relatives, sterilizations due to certain parental conditions, and outside evaluation when help is requested from the health or social care services, i.e. in cases of assisted reproduction or adoption. But once the woman or couple has been deemed eligible for childbearing in general, the number of children is not regulated by law. By contrast, the Western moral opinion is divided on the issue of a woman's right *not *to have children. The Roman Catholic Church opposes "artificial" contraception, and induced abortions are a hot political and moral issue in many countries. Abortions are clearly in the political arena, and individual women's choices are often regulated. A common legal arrangement is that abortions are allowed if performed in early pregnancy by licensed health professionals [1].

China has a national policy regulating the number of children that a woman may have. The goals of population policy and resulting fertility have been extensively described in the English-language literature [2-7], but descriptions of how the policy has been implemented are few and patchy. A crucial and innovative element has been that of "official permission to be pregnant". In most other countries these types of permission are only socially constructed. The central concept applied at the individual level is that of "illegal pregnancy" (in Chinese "ji hua wai ren shen", "unauthorized" pregnancy). We use the term "illegal pregnancy" in analogy with that of illegal abortion. "Unplanned pregnancy" is misleading to non-Chinese readers, because this term is commonly used in reference to planning by individual women, not to permission given by authorities. "Unauthorized" or "unapproved" pregnancy would be the most accurate translation, but it is not suggestive of the incentives and fines attached.

The purpose of this article is to describe and discuss the concept of illegal pregnancy and how it has been applied in practice. We will describe how it has been operationalized and how it relates to the sex distribution of newborns and to abortions. The concept will be examined from a historical perspective, after a brief description of China's population policy.

## Methods

Contents of the laws and regulations were obtained from the original documents, and from previous English-language (identified in the text) and Chinese-language articles. The interpretation of the regulations is based on our general knowledge and previous research on Chinese policies. In China, state plans and decisions are frequently passed via other structures than the law, and prior to 2001 most population policy documents were decisions and official letters from the party or civil administration, called regulations in the subsequent text. Many regulations were made at provincial level (30 provinces).

National empirical data derive from published reports and statistics, identified in the text. Local data are from published research articles or from our own research, during which we collected data on pregnancy outcomes of a trial involving the introduction of comprehensive prenatal care in a rural county in Anhui Province [8].

### China's population policies

Communist China was founded in 1949 after the Japanese occupation and a long and devastating civil war. Initially the Government instituted a strong pronatalistic policy encouraging child-bearing by means of child subsidies, and prohibiting contraceptives, abortions and sterilizations [9,10,2]. This policy was relaxed in 1953, and contraceptives and abortions under certain conditions became available. In 1957, as part of the government's first birth control campaign, legal access to abortion was made easier [11].

The dramatic peak in fertility observed in 1962 led the government to change its policy and family planning in densely populated urban areas was promoted. The Cultural Revolution halted this program, but in 1973 the first antinatalistic population policy was launched: overpopulation was considered a threat to modernization and development. The 1973 policy used approaches such as education to individual women and couples, provision of models of reproductive behavior, and improvement of contraceptive and abortion services [12]. The policy promoted delayed marriage, long intervals between births, and a two-child family.

The 1973 policy was strengthened in 1975 defining population growth targets and introducing locally-managed collective birth plans [13]. In 1979 a one-child policy, which previously already had been experimented in some provinces, was introduced to the whole country. In addition to the requirement of one child per couple it set national targets (population less than 1.2 billion and population growth zero in 2000). A new separate vertical service structure (family planning, FP) was formed to implement the policy and monitor it; until 1979 family planning had been a part of health care and was organized at the local level. In 1981, the State Family Planning Commission (currently called the National Population and Family Planning Commission, the equivalent of a "Population Ministry") was formed to coordinate the activities. In 1982, family planning, aiming at reducing population growth, was included in the Constitution as a basic state policy [2]. Since 1981, the marriage law has explicitly required couples to practice family planning [14]. The family planning policy did not become a national law until 2001, and each province produced its own regulations on the basis of national guidelines [15].

The one-child family policy led to forced family planning and opposition. This and the economic reforms of the early 1980s, which reduced the state's control, led to a more permissive policy in 1984 allowing modifications at the provincial level, usually around the rules of having a second child. In the late 1980s, clear provincial rules were demanded. The new regulations [16-27] focused, in addition to higher order births, on preventing early-age marriage and childbirth, and on reducing the high rate of induced abortions [28].

In the 1990s, population policy stressed stability, and only small modifications in population targets at the provincial level were made. Contraception was promoted instead of abortions, and emphasis was placed on improving regular services instead of conducting separate campaigns. High contraceptive use rates were achieved. The importance of integrating population policy into general social and economic planning was acknowledged, and the question of an aging population became an important consideration [29].

Currently, different provinces have different rules [30,2]. The commonest provincial rule for rural areas is "two children if the first is a girl". But some provinces have adopted "two children with a four-year interval" [2]. In the case of minority groups, more children are allowed. For cultural reasons very few children have been born outside marriage, and most women marry [31] and have their first child shortly after the marriage. Contraceptives are now available free of charge and are commonly used.

### Illegal Pregnancy

For a pregnancy to be "legal" (i.e. approved by secular authorities), the woman has to have permission for it from the population authorities. The family planning regulations and the 2001 law define legal (authorized) births, i.e. what is allowed [32], and "illegal" (unauthorized) pregnancy (or birth following such pregnancy) must be logically concluded as the opposite. The regulations stipulate incentives, but disincentives to avoid illegal pregnancies are defined at a lower level, mostly by county FP committees. Whether the pregnancy is legal depends on whether the pregnancy history of the parents qualifies them to have a (new) child, and whether the child quota laid down in the yearly plan of the village or workplace is full or not. The rules vary from one area and workplace to another. The process of determining legality may include the issuing of a formal certificate from the local family planning authority. The description from two urban areas [33] shows that getting the permission may be complicated and is not a formality.

The Chinese family planning regulations do not include the concept of an "illegal child" (i.e. a child born out of an "illegal pregnancy"), and the law prohibits discrimination against children born outside marriage [34]. However, children from illegal pregnancies may not be registered or treated equally until their parents pay the fines imposed as punishment. Especially in urban areas registration with the local authority is required for medical care, schooling and employment. The use of incentives and disincentives in the population policy implementation has varied by time and place, and it is likely their impact has declined over time due to the large social changes, such as growth of private housing in cities, shrinking of state sector enterprises and internal migration. One incentive from 1988 is the "one-child certificate" which is a contract between a couple and the local government. It gives parents who agree to have only one child certain economic rewards, such as a monthly stipend, free obstetric care, increased maternity leave, highest priority in education and health care for the child, preferential treatment when one is applying for housing, and a supplementary pension [15,30,35]. Disincentives for violators include losing housing and school benefits or having to pay higher fees and fines. Fines, currently called extra tax, may be substantial: according to one source, they amount to 10–20 % of a family's annual income; and according to another, they exceed the average annual income [35]. Qian [28] reports fines of 2.5 times the village's per capita income. Payment period and how actively the fines are collected have varied by place [15].

Enforcement of the population policy has been pursued via the Communist Party and the State Family Planning (FP) Commission, both of which have a functioning vertical structure. The FP structure extends downwards from the State FP Commission to province, city, district (urban) or county (rural), street/township, and finally to neighborhood FP / village FP committee. FP service centers at the county and township level have taken over some services previously provided by local health services [15]. The FP organization has good connections to women's voluntary organizations [35]. In the 1990s, there were an estimated 300 000 FP officials, and hundreds of thousands of part-time FP workers in villages, called "cadres" (lay-people working part time for local authorities) [2]. Parallel to the hierarchical structure of civil servants, there is the party organization, and the leaders of the party at each level share the decision making with leaders of the civil servant departments. Cadres are usually also members of the Communist Party.

Implementation of the family planning policy has been taken seriously and extensive and ongoing ideological education activities involving authorities and cadres were introduced. Education has been provided on both, general and individual level. Individual women are persuaded to comply with the rules, as care and control are provided by the same individuals [33]. Educational programs address the general good: the importance of the population policy for the society. Yearly population plans, including numbers of births and the organization of FP services, are developed by local authorities, and state and municipal workplaces. Data collection is organized by cadres. Villages in rural areas and workplaces in urban settings take collective responsibility, and sign a contract to make the plan for targets and enforcement [33,36].

In rural areas, an extensive system has been created at the village and district level to ensure constant surveillance of contraceptive use and pregnancy status of all married women at reproductive age. It is common for married women to be requested to visit an FP station every two or three months for pregnancy testing, allowing for early pregnancy detection.

In cities, family planning officials and cadres within workplaces have a central function. The surveillance of contraceptive use may be more common than surveillance of pregnancies, as fear of loosing a job may motivate women not to have an illegal pregnancy [33]. Alongside education, accessible services, incentives, disincentives, and the surveillance of all married women at reproductive age, a "personal responsibility system" was introduced in the early 1990s to guarantee that the local birth plan is followed [15,36,35]. Under this system, the heads of both the FP and Party committees at each level are personally responsible for ensuring that the targets are met and the rules are followed. If deviations occur, the officials may be subject to penalties, including the withholding of monetary bonuses, the loss of promotion and dismissal. This has resulted in false reporting and financial negotiations with an official's constituency to compensate for financial losses [15,36].

### Abortions and sex ratio

Until 1953, induced abortion was available only on the basis of protecting a woman's or her existing children's health; in addition abortions had to be performed early and a physician's certificate was required [11]. In 1957, abortion was made available at a woman's request within the first 10 weeks of pregnancy and each woman was allowed one abortion per year. The 1979 abortion law abolished most restrictions, and set 28 weeks of gestation as the upper limit for legally performing abortions [37]. Some provinces have made their own laws stipulating the place and performer of the abortion [12].

Whether a woman ends up having an abortion depends on the strength of her and her family's wish to have a further child, and her acceptance of abortion and the population control norms. It seems that the population at large is permissive towards abortion [38] (Table 1). In our study, conducted in a rural setting in Anhui Province, the outcome of pregnancies varied considerably according to the legal status of the pregnancy [8].

**Table 1:** Recorded induced abortions in China, 1975–1999

	Induced abortions^1)^	Live births^1)^	Ratio
1976	475	1853	0.26
1978	539	1745	0.31
1980	953	1779	0.54
1982	1242	2238	0.56
1984	889	2055	0.43
1986	1158	2384	0.49
1988	1268	2457	0.52
1990	1349	2391	0.56
1992	1042	1759	0.59
1994	947	2104	0.45
1996	883	2067	0.43
1998	738	1991	0.37

^1) ^in 10 000

Source: Ref. 40.

The number of abortions increased rapidly after the introduction of the 1979 population policy. Some reports suggest that women may have been forced to have an abortion if an illegal pregnancy occurred [39]. Applying pressure to undergo sterilization was apparently even more common [40]. However, there are reports that forced abortions are uncommon nowadays [40-43]. The temporary concealment of pregnancies and children lessens the pressure of having an unwanted abortion. Hidden children are usually registered later, sometimes as immigrants.

An unnatural sex ratio at birth, i.e. more baby boys than girls, has been reported from China at least since the early 20^th ^century. The excess of male babies declined considerably between 1936 and 1960 [44], and increased from the mid-1970s onwards [45,44,46]. The sex ratio at birth is higher in rural than in urban areas [46,47], and the variation by province is large [34,44,48]. It has been shown that the higher parity is, the higher is the sex ratio, especially if there are no previous sons [34,49,50]. The unnatural sex ratio at birth can be the result of sex-selective abortions, hiding and/or not reporting baby girls, or of extra deaths among newborn girls [8,34,33,38]. Previously hiding of baby girls might have been the main cause for the high sex ratio at birth, as indicated by a higher numbers of girls at later ages as compared to births. However, as shown in our study in one rural county in Anhui province [our unpublished data], sex selective abortions may have become an important reason for the high sex ratio at birth.

Fetal sex determination is illegal in China and health professionals performing it are penalized [34,38]. The first regulation against fetal sex determination was issued by the national health ministry in 1989, and the content repeated in the 1995 law on maternal and child health care. The 2002 FP law explicitly refers to the ban of using ultrasound and other technologies to determine fetal sex. Abortions on the basis of a child's sex are illegal, and if detected the parents' right to have a second child is lost. Ultrasound scanning is now widely available [34,38], and the various regulations against its use for fetal sex determination indirectly hint that such use has occurred. Many other Asian countries and regions, including South Korea, Taiwan, Hong Kong, Singapore and Thailand have experienced a remarkable decline in fertility from the 1950s to 1990 [see e.g. [51,9]]. With the exception of Singapore, these countries had no other explicit population policies except for the promotion and wider provision of family planning services (Table 2).

**Table 2:** Comparison of sex ratios in China, Taiwan, and the South Korea

year	Fertility (TFR)			Sex ratio		
	
	China	Taiwan	South Korea	China	Taiwan	South Korea
1981	2.6	..	..	107	107	107
1982	2.9	..	2.7	107	107	107
1983	2.4	2.2	..	108	107	108
1984	2.4	..	2.1	109	107	109
1985	2.2	..	..	111	107	110
1986	2.4	1.7	..	112	107	112
1987	2.6	1.7	1.6	111	108	109
1988	2.5	1.9	1.6	108	108	114
1989	2.4	1.7	..	111	109	112
1990	2.3	1.8	1.6	115	110	117
1991	2.2	1.7	..	116	110	113
1992	2.0	----	..	114	-----	114
1995	1.8	----	1.7	117	-----	113
2000	1.7^1)^	----	1.5	117	-----	110

Source: Ref. 66, 67, 68, 69.

1) own estimate from the 2000 census.

## Discussion

The concepts of illegal pregnancy and illegal birth do not exist in the Western literature. Pregnancies may be unwanted or not socially acceptable, or they may not be considered suitable for a particular woman's role, but they have the same legal status as other pregnancies. Even pregnancies starting with an illegal act, e.g. sex involving under-aged women or rape, are legal. In China, the concepts of "illegal" (unauthorized) pregnancy and birth are not mentioned in official Chinese documents, and they have to be deduced from definitions concerning what is "legal" (authorized). These concepts have not been analyzed in the English-language literature, where euphemisms such as "unplanned pregnancy" have been used to describe the Chinese situation. This causes confusion, because planning of pregnancies and births is understood in the West as being a private matter, while in Chinese literature planned pregnancies and births refer to those "approved by family planning authorities".

Previously, in Western countries, the concept of an "illegal" or "illegitimate" child was used. In older Finnish statistics, children born out of wedlock were classified as "illegitimate" [52-61]. We searched for literature from Finland to see whether some pregnancies and births had been considered illegal. This was not the case, even though sex before marriage or with a person other than one's legal partner had been a criminal offense from the end of the 17^th ^century until 1926 (premarital sex) and 1948 (extramarital sex). Pregnancy and a child born as the result of a criminal act were protected, and abortion and infanticide were strictly condemned. "Illegitimate" children had otherwise the same legal rights than other children, but only in 1975 they were granted the same paternal inheritance rights as other children.

In the 20^th ^century in Finland, the regulation of pregnancy in terms of who may be pregnant, dealt with the presumed health (physically or mentally) of the child to be born. The earlier methods to prevent women with certain handicaps or assumed inheritable diseases from conceiving, such as restrictions on marriage, were replaced later on by sterilizations and abortions [62]. With the new techniques for identifying fetal characteristics, interest in selective abortions has increased, but the modern fetal screening methods and selective abortions are voluntary, emphasizing the parents' freedom of choice.

### Is Chinese population policy unique?

Our answer is no and yes. No, considering the fact that the distinction between illegality and strong social disapproval or lack of choice is not a major one, particularly among the poor. Both situations mean lack of choice, particularly if incentives not to have children and disincentives to have them are used. In countries in which religion is a strong social factor, from an individual's point of view the distinction between morals and laws may be hazy, and social and legal control may be hard to distinguish from one another. Population targets, widespread family planning information, easily accessible services and increased sex ratios at birth, are not unique to China. The increased sex ratio in China was not intended, and the comparison with some other Asian countries suggests that when fertility is low in societies favoring men, sex ratios are likely to be high [63].

But yes – China's population policy is distinctive in two ways. Firstly, it has created an efficient system for the regular monitoring of all married women's pregnancy status. This allows early intervention by means of abortion, and constant enforcement of contraception. Secondly, it was designed to apply to all women. Usually, when a society has used legal measures to prevent individual women and couples from having children, it has acted selectively. Grounds have included the assumed health of the future child or the childbearing capabilities of the parents, especially that of the mother. In Europe, the largest group of women to be excluded from childbearing used to be non-married women. Currently, the largest group is very young women.

### Has the Chinese one-child policy been morally wrong?

The answer depends on one's view of who should decide on reproduction. Should it be freely decided upon by an individual / by a woman or whether a society or state should decide. The individualistic approach has been promoted to give women a choice. The proponents of individualism mainly live in countries in which large numbers of births have not been a societal problem. European and North American countries have had pronatalistic population policies, if any. In Western countries, terminating a pregnancy is legally controlled to a greater extent than being pregnant, although abortion laws vary considerably from one country to another [1].

When a society or its leaders feel there are too many people for the society to function well, moral judgment of actions undertaken to restrict the size of the population is influenced by the quality of implementation tools and the grounds for deciding that there are too many people. After 1979, the tools for restricting population size in China did not respect individuals, and at least in some places they were coercive. However, the worst forms, including forced abortions, are believed to have been rare [15]. The current national population policy explicitly prohibits coercion [40].

Many Westerners, with their cultural emphasis on gender equity, find sex-selective abortions disagreeable. To our knowledge there are no large-scale studies on Chinese views regarding sex-selective abortions. But a small study by Chu [38] found that even though rural women were undergoing such abortions, they did not think this was right and felt that it represented unfair treatment of girls.

### Has the Chinese one-child policy been wise?

Because it is unknown what would have happened without it, it is difficult to judge. When comparing the development in China to that in South Korea in the 1970s, the comprehensive and resource-intensive one-child family population policy in China did not seem to have been fully successful – the decline in birth rates was not exceptionally large. The 1979 reform of population policy coincided with the rural economic reform, with its shift from collective to private farming ("household responsibility"). This reform may have partly contradicted the aim of restricting and monitoring population growth [51,64,2]. The rapid changes in population policy may have confused people and lessened public support. But the aim of ensuring equity in the policy may have softened the criticism: all women and couples are faced with equally harsh requirements. Nathansen's [33] study from an urban area showed that most women resigned to the one child policy, because they accepted the reasons for it, even though their personal wishes may have been different. However, variations in local application and fines have created geographical and social inequity. Yearly fluctuation and unbalanced growth in different decades and generations puts an extra burden on the provision of services such as schools and child care facilities, and social security in old age. The system of personal responsibility, under which meeting the family planning targets is one central achievement indicator for civil servants at different levels, has worsened the quality of population statistics, thus impairing monitoring and predictions.

Sex-specific abortions have been practical in helping families to have at least one son with fewer births. In the short term both, parents and family planning personnel have benefited from this. But in the future, the social implications of fewer women may be considerable in a culture that values marriage. Lack of women is likely to have an impact on prostitution and trafficking of women, and other social problems [50].

This article has mainly analyzed the situation in China as it was up to 2000. China is rapidly changing, and this also applies to its population policy. Since 1994, the population policy has been reoriented. The new official policy emphasizes on quality of services, respect for the individuals, the needs of citizens, and informed choice [65]. Reproductive health rather than population control is promoted, as well as cooperation between the family planning system and the health sector. The 2002 FP law prohibits discrimination of baby girls or their mothers. All over the world governments employ various mechanisms to regulate reproduction. Traditional methods of state control include marital laws, making infanticide illegal, protecting children from negligence, and regulating sterilizations and abortions in various ways. Reproduction is socially regulated through religion, morals, values, and laws. Modern technology, with its effective contraceptive methods, possibilities to see and study the fetus, and easier and safer abortion techniques, has made the regulation of childbearing technically easier. A comparison of China, where the state regulates who can be pregnant and, for example, Ireland, where the state regulates who can have an abortion shows how relative norms are in the area of reproduction [1,37]. The case of China leads us to believe that at the societal level there is no shared "world" view on the legal control of reproduction. Whether such a view exists in the context of the values and morals of individual women or families is not a topic of this paper.

## Competing interests

The author(s) declare that they have no competing interests.

## Authors' contributions

EH analyzed the data and had the main writing responsibility.

ZW collected the Chinese language literature, analyzed it and commented the drafts.

GC helped in describing the Chinese system, helped in location the relevant literature, and commented the drafts.

KV participated in interpretation the findings and writing the article.

All authors read and approved the final manuscript.
